# Newly Diagnosed Atrial Fibrillation Indicators in Cryptogenic Stroke Survivors' P‐Wave Indices: A Systematic Review and Meta‐Analysis

**DOI:** 10.1002/joa3.70209

**Published:** 2025-10-22

**Authors:** Haikal Balweel, Surya Sinaga Immanuel, Jordan Budiono, Fransiskus Xaverius Rinaldi, Yeziel Sayogo, Novaro Adeneur Tafriend, Agus Harsoyo

**Affiliations:** ^1^ Department of Cardiology Gatot Soebroto Army Central Hospital Jakarta Indonesia; ^2^ School of Medicine and Health Sciences Atma Jaya Catholic University of Indonesia Jakarta Indonesia

**Keywords:** atrial fibrillation, cardiac conduction system disease, cryptogenic stroke, electrocardiography, P‐wave indices

## Abstract

**Background:**

Atrial fibrillation (AF) is the most common arrhythmia and a major cause of ischemic stroke recurrence. Cryptogenic stroke (CS) survivors face a higher risk for newly diagnosed AF (NDAF), with subtle ECG markers—prolonged P‐wave duration (PWDur) and increased P‐wave dispersion (PWDis)—potentially serving as early indicators. We aimed to quantify baseline PWDur and PWDis differences between CS survivors with NDAF and those in sinus rhythm.

**Methods:**

Following PRISMA guidelines, we systematically searched nine databases through January 2025 for observational studies assessing baseline P‐wave indices in adult CS patients in sinus rhythm. Data extraction and risk of bias assessment using the ROBINS‐E tool were performed independently. Pooled mean differences (MD) with 95% confidence intervals (CI) were calculated using a random‐effects inverse‐variance model. Subgroup/sensitivity analyses were conducted by age groups, male proportion, region, follow‐up duration, percentage of comorbidities, AF definition and detection methods, and ECG parameter. Heterogeneity was assessed using Cochran's *Q*, *τ* ^2^, and *I* ^2^ and interpreted using Cochrane thresholds; certainty was appraised with GRADE.

**Results:**

Ten studies, encompassing 1508 patients (mean age 66.72 ± 13.74 years; 55.7% male), met the inclusion criteria. Baseline PWDur was longer in the NDAF (MD 6.36 ms, 95% CI: 0.69–12.03, *p* = 0.03, *I* ^2^ = 73%, GRADE: moderate). Sensitivity analysis confirmed the robustness. PWDis remained non‐significant.

**Conclusion:**

Prolonged baseline PWDur may serve as a non‐invasive marker of atrial conduction abnormalities and a predictor of AF in CS survivors. Larger prospective studies are needed to validate its role in risk stratification and secondary stroke prevention.

**Trial Registration:**

PROSPERO: CRD42025646135

## Graphical abstract

This study found that baseline P‐wave duration is significantly prolonged in cryptogenic stroke survivors with newly diagnosed atrial fibrillation, supporting its role as a non‐invasive marker of atrial conduction abnormality and a tool for risk stratification and secondary stroke prevention.

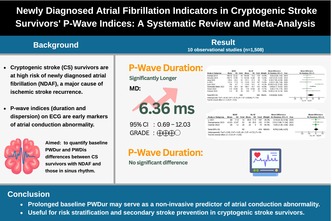

## 1 Background

Atrial fibrillation (AF) is the most common sustained arrhythmia, affecting millions globally and significantly increasing the risk of acute ischemic stroke and stroke recurrence [1, 2, 3]. Recent epidemiological data reveal that the incidence of AF has risen substantially in recent years, and patients with a history of stroke are particularly vulnerable to recurrent events [4, 5]. Given the high morbidity and mortality associated with recurrent strokes, early identification and management of AF in post‐stroke patients is a clinical imperative [6, 7].

Electrocardiographic (ECG) parameters, notably P‐wave duration (PWDur) and P‐wave dispersion (PWDis), have been extensively studied as markers of atrial conduction abnormality [8, 9]. PWDur is the time from the earliest onset of P‐wave activity to its last activity in a given lead. In contrast, PWDis is calculated by subtracting the minimum P‐wave duration from the maximum duration observed. Prolonged PWDur reflects delayed atrial conduction and structural remodeling, while increased PWDis indicates heterogeneity in atrial conduction—both conditions predisposing to AF [10, 11]. Numerous studies have demonstrated a significant association between these P‐wave indices and the incidence of AF in various clinical settings [12, 13, 14].

Although meta‐analyses have explored the relationship between P‐wave indices and AF in the general population, there is a paucity of evidence specifically addressing this association in cryptogenic stroke (CS) survivors [15]. CS patients represent a high‐risk group in whom subtle ECG abnormalities may provide early indicators to the development of newly diagnosed AF (NDAF) [16]. Identifying such differences in baseline P‐wave indices could have important implications for timely intervention and secondary stroke prevention.

This systematic review and meta‐analysis aims to assess the mean differences in baseline PWDur and PWDis between CS survivors who develop NDAF and those who remain in sinus rhythm. By rigorously synthesizing the available evidence, this study seeks to clarify the role of these ECG markers in stratifying risk among CS patients, thereby contributing to improved clinical decision‐making and patient outcomes. Furthermore, identifying baseline ECG differences in conjunction with atrial structure/function (e.g., left atrial volume index, left atrial strain, or cardiovascular magnetic resonance‐fibrosis) may refine risk stratification and secondary prevention in CS survivors.

## 2 Methods

This systematic review was conducted in accordance with the 2020 PRISMA (Preferred Reporting Items for Systematic Reviews and Meta‐Analyses) guidelines (Table S1), and the protocol was registered with PROSPERO [17].

### 2.1 Database and Literature Search

On January 16, 2025, we conducted a comprehensive literature search across several electronic databases, including ProQuest, PubMed, Europe PMC, Google Scholar, SAGE Journals, Science Direct, Wiley Online Library, EBSCOhost, and the Cochrane Library. Our search strategy employed a combination of relevant keywords and Medical Subject Headings (MeSH) terms such as “atrial fibrillation,” “cryptogenic stroke,” “P‐wave indices,” “P‐wave duration,” and “P‐wave dispersion.” Boolean operators (AND/OR) were used to refine and optimize the search results (Table S2). We applied filters to include only peer‐reviewed articles published in English from the inception of each database until the search date. In addition, the reference lists of pertinent reviews and articles were systematically screened to identify any further relevant studies.

### 2.2 Eligibility Criteria

Eligible studies will include observational research designs—specifically, cohort studies—focusing on adult patients with CS who were in sinus rhythm at baseline. To be included, studies must examine the association between baseline P‐wave indices, particularly PWDur and PWDis, and the subsequent development of NDAF during follow‐up. Studies will be excluded if CS is not the primary focus, if they lack clearly defined diagnostic criteria for CS or standardized methods for measuring P‐wave indices, or if they do not include a comparator group of CS survivors who remain free of NDAF.

### 2.3 Study Selection and Data Extraction

Three independent reviewers (SSI, JB, and FXR) meticulously screened the titles, abstracts, and full texts of all identified studies. Discrepancies were resolved through discussion with senior authors (HB, NAT, and AH). Data were systematically extracted to capture essential study characteristics, including study design, country, follow‐up duration, and diagnostic criteria for CS and AF. Specific outcome measures—namely, baseline PWDur and PWDis—were recorded for both the NDAF group and the no AF group. Additional data such as sample size, participant demographics (age, gender distribution), and relevant comorbidities (e.g., hypertension, diabetes, coronary artery disease) were also documented. Duplicate entries were identified and removed using EndNote X9's duplicate detection tool (Clarivate Analytics, Philadelphia, USA).

### 2.4 Risk of Bias Assessment

Three investigators (SSI, JB, and YS) independently assessed the risk of bias in all included studies using the ROBINS‐E tool (Risk of Bias In Non‐randomized Studies—of Exposures) in accordance with Cochrane Handbook recommendations [18]. The evaluation covered seven domains: (1) confounding, (2) bias in measuring exposures, (3) bias in participant selection, (4) bias from post‐exposure interventions, (5) bias due to missing data, (6) bias in outcome measurement, and (7) selective reporting bias. Each domain was assigned a risk level of “low risk,” “some concerns,” “high risk,” or “very high risk.” Any discrepancies were resolved through consultation with the senior authors (HB, NAT, and AH.).

### 2.5 Data Synthesis and Statistical Analysis

Statistical analyses utilized a random‐effects inverse‐variance model alongside the inverse‐variance method for continuous outcomes. The pooled mean differences (MD) with a 95% confidence interval (CI) were calculated for both PWDur and PWDis, with a *p*‐value of less than 0.05 considered statistically significant. The *τ* ^2^ values, *I* ^2^ statistic, and Cochran's *Q* test were used to assess statistical heterogeneity. A statistically significant result was determined as a Cochran's *Q* test *p* ≤ 0.10, and thresholds for *I* ^2^ were 25%, 50%, and 75%, respectively, to indicate low, moderate, and high heterogeneity [19, 20]. The certainty of evidence for each outcome was assessed using the Grading of Recommendations Assessment, Development, and Evaluation (GRADE) approach, which classified it as high, moderate, low, or very low certainty [21]. Publication bias and meta‐regression were evaluated when a minimum of 10 papers were available, adhering to guidelines to guarantee sufficient power for identifying bias and dependability [22].

We conducted several subgroup analyses to explore possible explanations of heterogeneity. We subgrouped by age groups (≥ 65 years old vs. < 65 years old), male proportions (≥ 55% vs. < 55%), percentage of comorbidities (hypertension [HT] [≥ 70% vs. < 70%] and diabetes mellitus [DM] [≥ 25% vs. < 25%]), and methodological factors, including follow‐up duration (≥ 18 months vs. < 18 months), regions (Asia vs. non‐Asia), AF episode definition (explicit ≥ 30 s threshold vs. other or unspecified), and detection methods (implantable loop recorder [ILR] vs. non‐ILR), and ECG parameters (paper speed 25 mm/s vs. 50 mm/s). The cutoffs were determined a posteriori, based on the distribution of the included studies. Sensitivity analyses were performed to evaluate the robustness of the original findings. Three supplementary procedures were implemented: (1) excluding studies with a high risk of bias, (2) applying the standardized MD (SMD) methodology, and (3) performing a leave‐one‐out sensitivity analysis. All statistical computations were performed using Review Manager (RevMan) version 5.4 (Cochrane, London, UK).

## 3 Results

### 3.1 Study Selection

**FIGURE 1:**
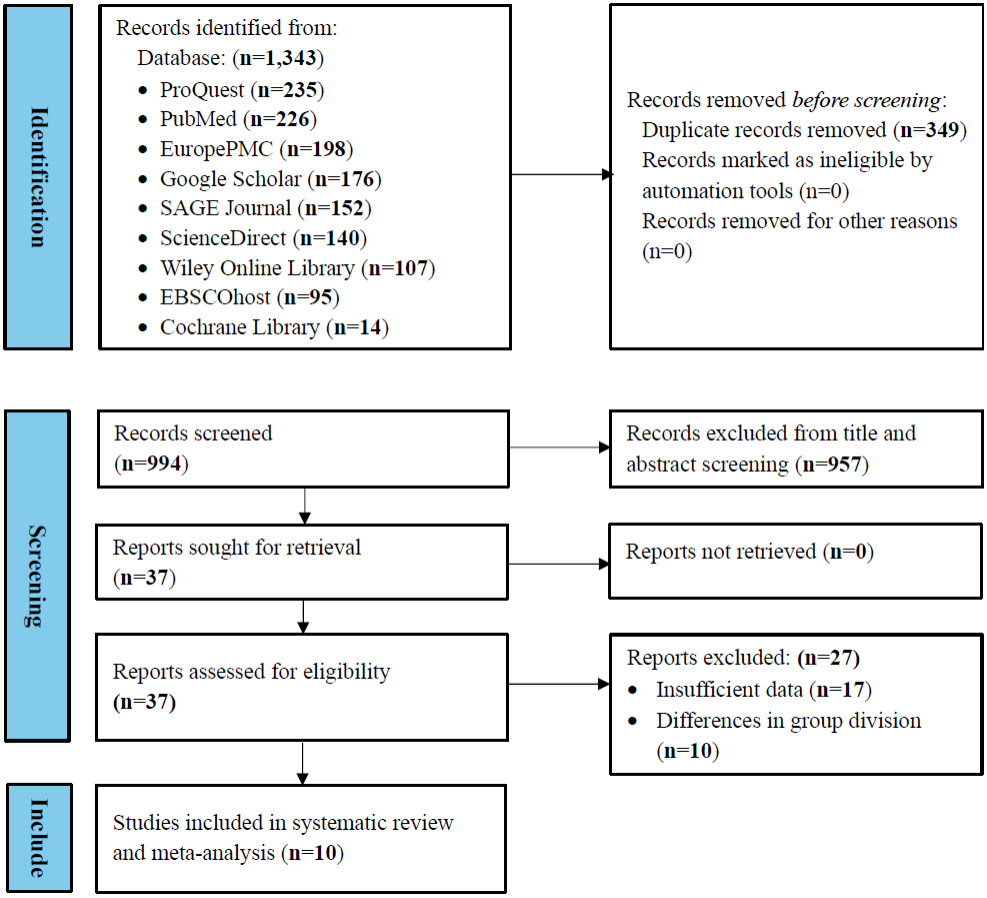
PRISMA flow diagram of study selection.

A systematic search yielded 1343 records from nine electronic databases (ProQuest [235], PubMed [226], Europe PMC [198], Google Scholar [176], SAGE Journals [152], Science Direct [140], Wiley Online Library [107], EBSCOhost [95], and the Cochrane Library [14]). After removing 349 duplicate records, 994 unique studies underwent title and abstract screening, which led to the exclusion of 957 records that did not meet the inclusion criteria. Of the remaining 37 studies selected for full‐text review, 27 were excluded—13 for insufficient data and 14 due to heterogeneous group classifications (e.g., Acampa et al. [2019] compared PWDur groups defined as > 40 ms vs. < 40 ms rather than segregating NDAF from non‐AF cases) [23]. Ultimately, 10 studies fulfilled all eligibility criteria and were incorporated into the meta‐analysis (Figure 1). Notably, eight studies examined the association between PWDur and the incidence of NDAF in post‐CS patients, while three studies evaluated the relationship involving PWDis.

### 3.2 Characteristics of the Studies

This study comprised 10 single‐center studies—eight retrospective investigations and two prospective cohort studies—published through February 2025 [24, 25, 26, 27, 28, 29, 30, 31, 32, 33]. The pooled sample consisted of 327 patients in the NDAF group and 1181 in the no AF group, with a mean age of 66.72 ± 13.74 years and a male predominance (55.7%). Geographically, the studies were distributed as follows: Germany (3 studies, 18.2%), Asia (3 studies, 36.5%)—including Korea (8.3%), Singapore (12.0%), and Thailand (16.2%)—Italy (1 study, 14.7%), America (1 study, 11.8%), Canada (1 study, 3.2%), and Norway (1 study, 15.6%). Details of the study and patient characteristics are summarized in Tables 1 and 2.

**TABLE 1:** Study characteristics and the criteria of cryptogenic stroke, PWDur/PWDis, and AF in each included study.

Study	Design	Database	Country	Period	Follow‐up (months)	Cryptogenic stroke criteria	PWDur/PWDis measurement	AF diagnostic methods
Sieweke (2019) [24]	Prospective cohort	Local certified Stroke Unit Hannover, Medical School, Hannover, Germany	Germany	August 2016–April 2017	0.08 ± 0.05	Strokes with vascular cause were discriminated according to the TOAST criteria. ESUS was defined as proposed by the Cryptogenic stroke/ESUS International Working Group	No definition or measurement method was provided	AF was defined as any episode detected by either 12‐lead ECG or 72‐h Holter monitoring in stroke patients using two‐channel (five‐lead) Holter‐ECG‐monitoring (GE Healthcare SEERTM 1000, Great Britain)
Acampa (2019) [25]	Retrospective cohort	Stroke Unit 41 of Siena University Hospital Italy	Italy	NA	60, 0	TOAST criteria for cryptogenic stroke	PWDur was measured from the beginning of the P‐wave deflection from the isoelectric line to the end of the deflection returning to isoelectric line in all simultaneous 12 leads	When normal sinus rhythm was replaced with irregular tachycardia lasting > 5 min with no visible P‐wave or with unorganized F wavelets as recorded on 7‐day ECG monitoring (25 mm/s and 10 mV/cm)
Jung (2020) [26]	Retrospective cohort	Chung‐Ang University Hospital (Seoul, Korea)	Korea	April 2015–February 2018	19.20 ± 9.60	TOAST criteria for cryptogenic stroke	PWDur assessed by signal‐averaged electrocardiography, is an established and precise method for evaluating inter‐ or intra‐atrial conduction delay and has predictive value for AF under different circumstances	Any episode of AF detected during follow‐up using 12‐lead ECGs during hospital stay; 24‐h Holter ECG monitoring; and 72‐h telemetry on the stroke unit with manual analysis
Marks (2020) [27]	Retrospective cohort	Cooper University Hospital in Camden, NJ	America	September 2015–December 2017	13.71 ± 7.81	TOAST criteria for cryptogenic stroke	PWDur were measured using the MUSE editor program and computerized calipers at an ECG rate of 50 mm/s and a voltage up to 10 mV/mm.	Any episode of AF detected by the ILR with a duration of at least 30 s using ILR (Medtronic Reveal LINQ TM or Medtronic Reveal XT LINQ TM), measured using the MUSE editor and computerized calipers at an 50 mm/s and 10 mV/mm
Li (2020) [28]	Retrospective cohort	Stroke unit at a Tertiary Care Hospital, Singapore	Singapore	October 2014–October 2017	25.20 ± 12.48	Cryptogenic Stroke/ESUS International Working Group criteria	The PWDur were measured in both lead V1 and II/It was defined as the time from the earliest onset of P‐wave activity in the lead to the last P‐wave activity in this lead PWDis was calculated by subtracting the minimum PWDur from the maximum PWDis	Any episode of AF detected during follow‐up using ILR (Medtronic Reveal LINQ) or 12‐lead surface ECG, measured manually in a digital format, at a 25 mm/s and 10 mm/mV
Leon (2022) [29]	Retrospective cohort	Kingston Health Science Center (KHSC)	Canada	January 2013–September 2019	16.00 ± 14.00	Cryptogenic stroke was retrospectively identified from medical records, with other stroke etiologies ruled out through standard work‐up	The PWDur was measured with semi‐automatic calipers.	Any episode of AF detected as recorded on ICM and 12‐lead ECG, measured semi‐automatic calipers and 25 mm/s, 10 mm/mV. Categorized to Morphology‐Voltage‐P‐wave duration ECG risk score.
Skrebelyte‐Strøm (2022) [30]	Prospective cohort	Akershus University Hospital, Lørenskog	Norway	May 2016–June 2018	29.69 ± 10.52	TOAST criteria for cryptogenic stroke	PWDur was defined as the longest P‐wave in any lead.	Episode of irregular heart rhythm without detectable P‐waves lasting more than 30 s using 24‐h Holter ECG (OxyHolter Recorder) (Maynard, MA, USA) and implantable cardiac monitors (ICMs), 12‐lead resting ECG, at a speed of 50 mm/s
Saengmanee (2023) [31]	Retrospective cohort	Maharaj Nakorn Chiang Mai Hospital	Thailand	January 2017–December 2021	0, 1	Symptomatic cerebral infarcts with no known plausible etiology after initial diagnostic evaluation.	Electrocardiographic parameters contained PWDis, which was measured from the surface ECG using WebPlotDigitizer version 4.6	New‐onset AF occurring during hospitalization and admission using 12‐lead ECG, inpatient cardiac telemetry
Höwel (2023) [32]	Retrospective cohort	TRACK‐AF prospective study	Germany	2010–2017	19.28 ± 2.94	TOAST 5b classification for ESUS, a subtype of cryptogenic stroke	PWDur was measured from the earliest to the latest atrial depolarization in any lead.	Any episode of AF detected with a duration of at least 30 s using ILR (Reveal XT, Medtronic, Minneapolis, MN, USA), 12‐lead ECG, measured manually, printed at a paper speed of 50 mm/s
Kreimer (2024) [33]	Retrospective cohort	University Hospitals St Josef Hospital and Bergmannsheil Bochum	Germany	September 2012–August 2020	23.11 ± 13.75	The diagnosis of ESUS was made by the neurologists after MRI imaging and exclusion of alternative causes.	PWDur was defined as the longest P‐wave in any lead.	Episodes of Irregular heart rhythm without detectable P‐waves lasting ≥ 30 s using ILR Medtronic (Reveal DX, Reveal XT, Reveal LINQ), St. Jude Medical (Confrm Rx), and Biotronik (BioMonitor 2‐AF, Biomonitor III). 12‐lead ECG at a rate of 50 mm/s and a volatage of 10 mm/mV

Abbreviations: AF, atrial fibrillation; ECG, electrocardiogram; ESUS, embolic stroke of undetermined source; ICM, implantable cardiac monitor; ILR, implantable loop recorder; MRI, magnetic resonance imaging; NA, not available; PWDis, P‐wave dispersion; PWDur, P‐wave duration; TOAST, Trial of org 10 172 in acute stroke treatment.

**TABLE 2:** Patients' characteristics and clinical parameters.

Study	AF types	Groups (*n*)		Age (years)^a^	Male (%)	HT (%)	DM (%)	CAD (%)	LAVI (mL/m^2^)^a^
Sieweke (2019) [24]	New‐onset, subclinical, and paroxysmal	AF	13	75.00 ± 9.70	61.5	100	30.8	5.4	NA
		Non‐AF	56	65.00 ± 13.70	66.1	57.1	19.6	23.1	NA
Acampa (2019) [25]	New‐onset, subclinical, and paroxysmal	AF	44	76.95 ± 7.59	43.1	55.0	14.0	6.0	NA
		Non‐AF	178	68.77 ± 13.65	50.0	59.0	21.0	13.0	NA
Jung (2020) [26]	New‐onset, subclinical, and paroxysmal	AF	32	70.90 ± 7.80	46.9	78.1	9.4	NA	NA
		Non‐AF	93	67.60 ± 13.20	58.1	66.7	20.4	NA	NA
Marks (2020) [27]	Subclinical and paroxysmal	AF	35	72.4 ± 11	48.0	91.0	29.0	11.0	NA
		Non‐AF	143	65.1 ± 11.7	50.0	77.0	41.0	15.0	NA
Li (2020) [28]	New‐onset, subclinical, and paroxysmal	AF	14	65.3 ± 11.9	57.1	85.7	35.7	7.1	36.6 ± 12.2
		Non‐AF	167	62.8 ± 12.4	71.9	73.7	35.9	18.6	27.0 ± 9.8
Leon (2022) [29]	New‐onset, subclinical, and paroxysmal	AF	7	58.50 ± 24.20	57.1	57.1	28.5	NA	31.4 ± 13.8
		Non‐AF	41	68.90 ± 10.90	53.6	65.8	48.7	NA	27.7 ± 7.8
Skrebelyte‐Strøm (2022) [30]	Subclinical	AF	84	72.10 ± 10.70	63.0	74.0	16.0	NA	42.0 ± 12.0
		Non‐AF	152	66.70 ± 13.00	61.0	57.0	11.0	NA	35.0 ± 9.0
Saengmanee (2023) [31]	New‐onset	AF	52	74.6 ± 10.6	36.5	71.2	36.5	11.5	NA
		Non‐AF	192	63.8 ± 14.5	56.0	59.9	29.7	6.8	NA
Höwel (2023) [32]	New‐onset	AF	20	71.28 ± 6.38	47.0	84.0	21.0	NA	30.2 ± 9.5
		Non‐AF	84	61.41 ± 15.09	59.0	72.0	24.0	NA	30.4 ± 9.0
Kreimer (2024) [33]	New‐onset, subclinical, and paroxysmal	AF	26	66.50 ± 9.60	73.0	85.0	31.0	12.0	NA
		Non‐AF	75	56.30 ± 11.10	56.0	67.0	19.0	7.0	NA
Summary^b^		1508		66.72 ± 13.74	55.7	71.8	26.1	11.4	30.30 ± 11.05

Abbreviations: AF, atrial fibrillation; CAD, coronary artery disease; DM, diabetes mellitus; HT, hypertension; LAVI, left atrial volume index; NA, not available.

^a^ Plus‐minus values are means ± standard deviations.

^b^ Accounting for only the available data.

### 3.3 Risk of Bias Assessment

**FIGURE 2:**
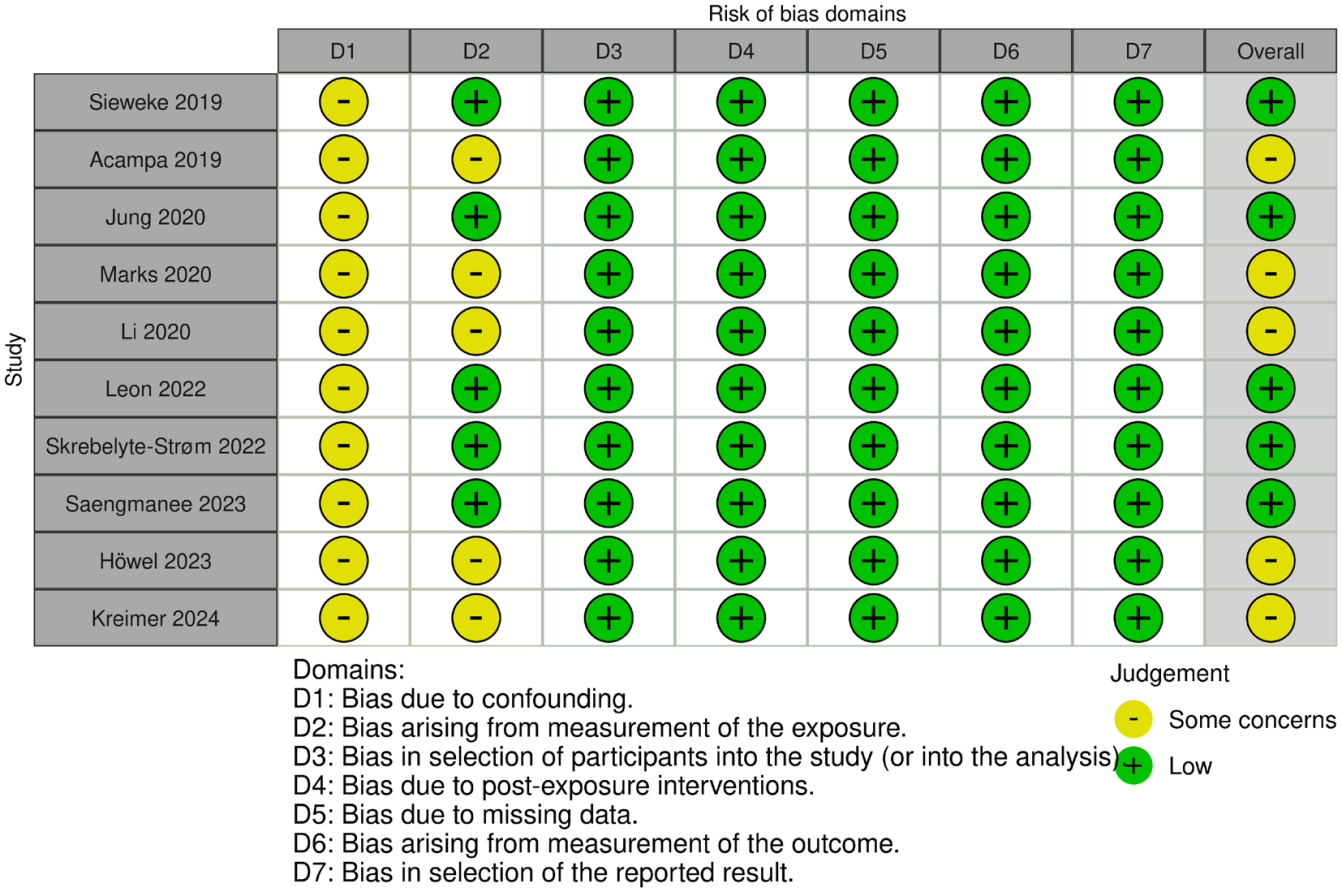
Risk of bias assessment using the Cochrane ROBINS‐E.

All included studies were evaluated using the Cochrane ROBINS‐E tool (Figure 2). Among the ten studies, five were classified as having a “low risk” of bias, while the remaining five had “some concerns.” Bias due to confounding was identified in all studies classified as having “some concerns,” primarily due to the lack of detailed information on stroke onset, which may have influenced the assessment of temporal relationships between atrial conduction abnormalities and AF detection [24, 25, 26, 27, 28, 29, 30, 31, 32, 33]. Additionally, five studies exhibited “some concerns” regarding exposure measurement, as they relied on ILR for AF detection rather than a Holter monitor. ILRs are highly sensitive for subclinical AF, but device algorithms can misclassify noise or other arrhythmias; manual adjudication mitigates this risk. We therefore classify potential misclassification as ‘some concerns’ where adjudication procedures were not fully detailed. Consequently, choosing an AF detection modality could have influenced the observed associations between P‐wave indices and AF in CS patients [25, 27, 28, 32, 33].

### 3.4 Meta‐Analysis of P‐Wave Duration in Cryptogenic Stroke Patients

**FIGURE 3:**
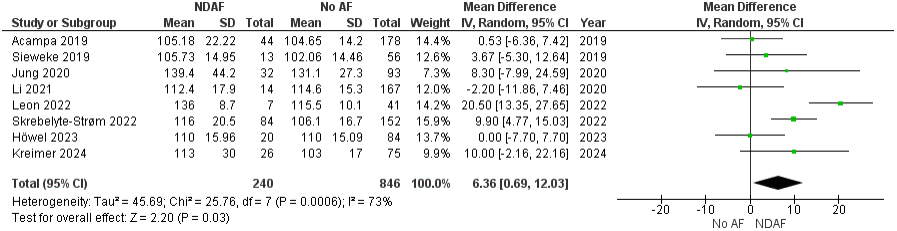
Forest plot of MD in PWDur between post‐CS NDAF and no AF groups.

Eight studies, encompassing 1086 CS patients, compared baseline PWDur between those who developed NDAF and those who remained free of AF [24, 25, 26, 28, 29, 30, 32, 33]. The pooled analysis demonstrated that PWDur was significantly longer in the NDAF group (MD = 6.36 ms, 95% CI: 0.69 to 12.03, *p* = 0.03) (Figure 3). Using the GRADE framework, the quality of evidence was rated as moderate, suggesting a reasonable degree of confidence in this modest effect size. However, there was notable heterogeneity among the studies (*I* ^2^ = 73%, *p* < 0.01) (Table 3). To aid clinical interpretation, we also examined the pooled absolute baseline PWDur values. The weighted mean PWDur was approximately 116.1 ms in the NDAF group and 110.5 ms in the group without AF.

**TABLE 3:** The GRADE approach on post‐CS NDAF compared to no AF in PWDur and PWDis.

Certainty assessment							Summary of findings				
Participants (studies) follow‐up	Risk of bias	Inconsistency	Indirectness	Imprecision	Publication bias	Overall certainty of evidence	Study event rates (%)		Relative effect (95% CI)	Anticipated absolute effects	
							With no atrial fibrillation	With newly diagnosed atrial fibrillation		Risk with no atrial fibrillation	Risk difference with newly diagnosed atrial fibrillation
*P‐wave duration*											
1086 (8 non‐randomized studies)	Not serious	Serious^a^	Not serious	Not serious	None	⨁⨁⨁◯ Moderate^a^	846	240	—	846	MD **6.36 ms more** (0.69 more to 12.03 more)
*P‐wave dispersion*											
526 (3 non‐randomized studies)	Not serious	Not serious	Not serious	Serious^b^	None	⨁⨁⨁◯ Moderate^b^	434	92	—	434	MD **0.79 ms more** (2.69 fewer to 4.27 more)

Abbreviations: CI, confidence interval; MD, mean difference.

^a^ Downgraded by one level due to substantial heterogeneity, which was not resolved by sensitivity analysis.

^b^ Downgraded by one level due to wide confidence intervals.

No studies were identified as having a high risk of bias for PWDur; therefore, the sensitivity analyses performed were SMD analysis and leave‐one‐out analysis, with results broadly consistent with the primary analysis. The SMD results are 0.34 with 95% CI: 0.04 to 0.63, *p* = 0.02 (Figure S1). A leave‐one‐out sensitivity analysis (Table 4) confirmed the robustness of these findings, with MD estimates ranging from 4.08 to 7.52 ms and *p*‐values spanning from 0.01 to 0.11, while heterogeneity levels varied between 38% and 77%. In most iterations, the effect remained statistically significant, although excluding certain studies led to borderline significance.

**TABLE 4:** Leave‐one‐out analysis of the MD in PWDur between post‐CS NDAF and no AF groups.

Sensitivity analysis				
Dataset excluded	MD	95% CI	*I* ^2^	*p* for effect
Sieweke (2019) [24]	6.74	0.32, 13.15	76%	0.04
Acampa (2019) [25]	7.34	1.12, 13.56	72%	0.02
Jung (2020) [26]	6.20	0.10, 12.29	77%	0.05
Li (2021) [28]	7.52	1.58, 13.47	73%	0.01
Leon (2022) [29]	4.08	−0.02, 8.19	38%	0.05
Skrebelyte‐Strøm (2022) [30]	5.70	−1.21, 12.61	75%	0.11
Höwel (2023) [32]	7.37	1.21, 13.53	73%	0.02
Kreimer (2024) [33]	5.94	−0.29, 12.17	76%	0.06

Abbreviations: CI, confidence interval; *I* ^2^, heterogeneity; MD, mean difference.

### 3.5 Subgroup Analysis P‐Wave Duration in Cryptogenic Stroke Patients

Cut‐off values were determined a posteriori based on the distribution of included studies and their clinical interpretability: Age ≥ 65 years old; Male ≥ 60%; HT ≥ 70%; DM ≥ 25%, along with methodological factors (follow‐up duration, region, AF episode definition, AF detection, ECG paper speed). None of the interaction tests showed a statistically significant subgroup difference (Age ≥ 65 years old vs. < 65 years old: MD 8.73 [1.43, 16.03] vs. 1.52 [−4.73, 7.77], subgroup difference = 0.14; Male ≥ 60% vs. < 60%: MD 5.82 [0.08, 11.56] vs. 7.27 [−3.85, 18.39]; subgroup difference = 0.82; HT ≥ 70% vs. < 70%: 1.52 [−4.73, 7.77] vs. 8.73 [1.43, 16.03], subgroup difference = 0.14; DM ≥ 25% vs. < 25%: MD 9.39 [−12.85, 31.63] vs. 4.99 [0.80, 9.19], subgroup difference = 0.70; Follow‐up duration ≥ 18 months vs. < 18 months: MD 4.09 [−0.77, 8.95] vs. 12.31 [−4.18, 28.80], subgroup difference = 0.35; Region Asia vs. non‐Asia: MD 0.92 [−8.49, 10.32] vs. 7.45 [0.99, 13.91]; subgroup difference = 0.26; AF episode definition ≥ 30 s threshold vs. other or unspecified: MD 6.53 [−0.37, 13.44] vs. 6.20 [−3.08, 15.48]; subgroup difference = 0.95; AF detection with vs. without ILR: MD 1.52 [−4.73, 7.77] vs. 6.20 [−3.08, 15.48], subgroup difference = 0.41; ECG paper speed 50 mm/s vs. 25 mm/s: MD 6.53 [−0.37, 13.44] vs. 6.45 [−7.97, 20.87], subgroup difference = 0.99) (Figures S2–S10).

Directionally, larger MDs were seen in older cohorts and those with lower HT prevalence and in non‐Asian settings, whereas associations were smaller in younger or ILR‐based cohorts; however, none of these differences reached statistical significance. Note that several subgrouping variables overlap across the same studies (e.g., age < 65 years, HT ≥ 70%, and ILR use), so these patterns should be interpreted as exploratory.

### 3.6 Meta‐Analysis P‐Wave Dispersion in Cryptogenic Stroke Patients

**FIGURE 4:**
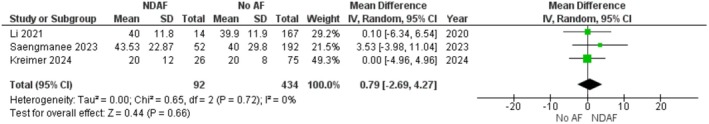
Forest plot of MD in PWDis between post‐CS NDAF and no AF groups.

Three studies, involving 526 CS patients, assessed the difference in PWDis between the NDAF and no AF groups [28, 31, 33]. The pooled analysis revealed a non‐significant increase in PWDis among patients with NDAF compared to those without AF (MD = 0.79 ms, 95% CI: −2.69 to 4.27, *p* = 0.66) (Figure 4). There was no evidence of heterogeneity (*I* ^2^ = 0%, *p* = 0.72), and no study was judged to be at high risk of bias. Therefore, only a sensitivity analysis using SMD was performed, while no subgroup analyses were conducted. The result was not significant (SMD = 0.07, 95% CI: −0.16 to 0.30, *p* = 0.66) (Figure S11). According to the GRADE approach, the certainty of the evidence was also judged as moderate, indicating a reasonable level of confidence in this modest effect size (Table 3).

## 4 Discussion

Our systematic review and meta‐analysis revealed that CS patients who develop NDAF exhibit a significantly prolonged baseline PWDur compared to those in the no AF group (MD = 6.36 ms, 95% CI: 0.69–12.03, *p* = 0.03). This finding, supported by moderate‐quality evidence per the GRADE framework, suggests that PWDur may be a useful electrophysiological marker for identifying CS survivors at risk of AF. Despite moderate heterogeneity (*I* ^2^ = 73%), SMD and leave‐one‐out analyses confirmed the direction and magnitude of the effect.

The test for subgroup interaction by ethnicity was non‐significant; the association was detectable in non‐Asian cohorts but not Asian cohorts, a difference most likely driven by the small number of Asian studies. Further subgroup analysis comparing ILR versus non‐ILR methods revealed no significant interaction. Exploratory subgroup analyses across demographic, clinical, and methodological factors did not identify any significant effect modifiers. Although certain directional trends were observed, these are likely confounded by overlapping variables and limited datasets, and thus remain exploratory rather than definitive. Because fewer than ten studies reported PWDur, meta‐regression was not feasible. By contrast, the pooled analysis of PWDis across three studies involving 526 patients did not show a significant difference between the NDAF and no AF groups (MD = 0.79 ms, 95% CI: −2.69 to 4.27, *p* = 0.66), with no heterogeneity detected (*I* ^2^ = 0%). These results underscore the clinical relevance of PWDur while highlighting the need for further research to elucidate the role of PWDis in this high‐risk population.

The apparent association between prolonged PWDur and NDAF in CS survivors indicates its value as a non‐invasive marker of atrial conduction abnormalities. Several large‐scale population‐based studies have established absolute PWDur as a robust predictor of incident AF. Notably, the Atherosclerosis Risk in Communities study, the Malmö Preventive Project, and the REGICOR cohort consistently demonstrated that a baseline PWDur of ≥ 120 ms is associated with a significantly increased risk of developing AF over time [9, 34, 35]. These findings underscore the role of prolonged atrial conduction as an early electrophysiological marker of atrial remodeling and susceptibility to arrhythmia.

The NDAF cohort's pooled mean baseline P‐wave duration was 116 ms (SD = 27 ms); thus, many—but not all—patients exceeded the conventional abnormal threshold (≥ 120 ms). This variability shows that a single 120‐ms cut‐off cannot capture every at‐risk individual; PWDur should be weighed alongside other clinical factors when selecting patients for extended rhythm surveillance. Therefore, these epidemiologic data not only validate PWDur as a general risk factor for AF but also support its application in targeted screening and secondary prevention efforts within the CS population.

In addition, signal‐averaged ECG (SAECG) studies have demonstrated markedly prolonged PWDur in CS patients (140 ± 16 ms vs. 123 ± 17 ms, *p* < 0.01), reinforcing that delayed intra‐ and inter‐atrial conduction, structural remodeling, and heightened arrhythmogenicity are already present in a subset of this population [36, 37]. These electrophysiological changes may signal an underlying atrial myopathy, a condition that renders patients vulnerable to cardioembolic events even in the absence of manifest AF. Recognizing these early conduction abnormalities in the CS population is crucial for timely intervention and secondary stroke prevention [38].

Although the mean inter‐group difference in our meta‐analysis was only 6 ms, large population cohorts report that every additional 10 ms of P‐wave prolongation beyond the normal range confers a 10%–20% relative increase in incident AF, while a 5–6 ms increment corresponds to an estimated 5%–10% risk rise [39]. Such seemingly small extensions become clinically meaningful when they shift a patient across the abnormal threshold—for example, from 114 ms to ≥ 120 ms—indicating inter‐atrial conduction block. Overall, even a slight extension of the P‐wave can serve as a relevant marker of atrial substrate abnormalities and heightened AF susceptibility, particularly in older adults or individuals at elevated cardiovascular risk.

In terms of clinical implications, in CS survivors, PWDur approaching or exceeding ~120 ms—particularly when accompanied by enlarged left atrial volume index (LAVI) or impaired left atrial strain on echocardiography—can help identify patients who merit prolonged rhythm monitoring (e.g., ILR) over short‐term surveillance [40]. Where available, cardiac magnetic resonance (CMR) assessment of atrial fibrosis may add complementary substrate information, acknowledging that ECG‐derived indices should complement—not replace—structural and functional markers [41]. This integrated, multimodal approach may better identify the subset of CS patients most likely to benefit from extended monitoring and early secondary prevention strategies.

In contrast, PWDis did not differ significantly between the NDAF and no‐AF groups, although a non‐significant trend toward higher values in the NDAF cohort was observed. This contrasts with the study by Peng et al., which reported a significant PWDis difference in patients with recurrent AF [42]; the discrepancy likely stems from differing populations (cryptogenic‐stroke survivors initially in sinus rhythm versus established AF) and the limited statistical power of our PWDis dataset. Larger, prospectively monitored cohorts are needed to clarify whether PWDis offers incremental prognostic value beyond PWDur in the CS setting.

Our meta‐analysis exhibits several notable strengths. We conducted an extensive literature search across multiple databases, reducing the likelihood of omitting relevant studies. Furthermore, applying the GRADE framework enabled a rigorous quality assessment beyond mere data pooling. We also executed subgroup analyses (Asian vs. non‐Asian ethnicity and ILR vs. non‐ILR monitoring) to explore potential sources of heterogeneity, and we re‐ran the main model using the standardized mean difference to confirm that the direction and magnitude of the effect were unchanged. A leave‐one‐out sensitivity analysis further confirmed the robustness of our findings despite considerable heterogeneity.

Nevertheless, several limitations warrant attention. First, the overall number of studies—particularly those evaluating PWDis—was relatively small, contributing to heterogeneity in effect estimates. Second, the predominance of retrospective designs among the included studies raises potential biases compared with prospective investigations. Third, variation in AF‐detection methods (e.g., ILR, Holter monitoring, single‐time‐point ECG) could have led to misclassification bias due to differing diagnostic sensitivities. Fourth, although subgroup analyses were performed, their power was limited, and meta‐regression could not be undertaken because fewer than ten studies reported PWDur. Fifth, considerable heterogeneity in PWDur outcomes (*I* ^2^ = 73%) persisted despite subgroup analyses, indicating that some clinical or methodological differences across studies may not have been fully captured. Variability in measurement techniques (manual, semi‐automated, or SAECG; or lead selection) likely contributed to this residual inconsistency, despite the overall consistency in effect direction. Subgroup analyses for LAVI and comorbidities, particularly CAD, were not performed due to incomplete data. These factors are therefore regarded as potential confounders, highlighting the importance of standardized and harmonized reporting in future prospective studies. Given that fewer than 10 studies reported PWDur, conducting a meta‐regression would be underpowered and potentially misleading; thus, we prioritized subgroup and sensitivity analyses. Finally, the clinical utility of P‐wave indices as biomarkers for NDAF in CS survivors remains constrained by the absence of standardized reference values that account for age, sex, and comorbidities.

Future research should focus on large, community‐based prospective cohorts to establish normative data for P‐wave indices. Integrating imaging modalities such as echocardiographic atrial‐strain analysis or cardiac magnetic‐resonance fibrosis mapping may further elucidate the pathophysiologic mechanisms underlying abnormal P‐wave indices, enhancing their reliability as screening tools. These efforts are essential to determine whether P‐wave indices can serve as independent biomarkers or complementary indicators for risk stratification in CS patients.

## 5 Conclusion

Our systematic review and meta‐analysis demonstrate that CS survivors who later develop NDAF have longer baseline PWDur than those who remain in sinus rhythm. PWDur may serve as a potential non‐invasive marker for atrial conduction abnormalities and a valuable tool for risk stratification in cryptogenic stroke survivors; however, given the modest number of studies, predominance of retrospective designs, and substantial heterogeneity observed—particularly in analyses of PWDis—alternative explanations such as measurement variability and residual confounding cannot be excluded. The findings should be generalized with caution. Future large‐scale, prospective, community‐based studies with standardized reference values for P‐wave indices, complemented by advanced imaging modalities, are warranted to confirm these preliminary observations and refine their clinical utility for risk stratification and secondary stroke prevention.

## Ethics Statement

The authors have nothing to report.

## Consent

The authors have nothing to report.

## Conflicts of Interest

The authors declare no conflicts of interest.

## Supporting information


**Data S1:** Supporting Information.

## Data Availability

The data that supports the findings of this study are available in the article and the Supporting Information of this article.
