# Supplementary material for: Newly Diagnosed Atrial Fibrillation Indicators in Cryptogenic Stroke Survivors' P‐Wave Indices: A Systematic Review and Meta‐Analysis
Source: J Arrhythm. 2025 Oct 22;41(5):e70209. doi: 10.1002/joa3.70209 (PMC12541236; doi:10.1002/joa3.70209)

**Supplementary Material**

**Supplementary Table 1:** PRISMA 2020 checklist.

| **Section and Topic** | **Item #** | **Checklist item** | **Location where item is reported** |
| --- | --- | --- | --- |
| **TITLE** | | |  |
| Title | 1 | Identify the report as a systematic review. | Title |
| **ABSTRACT** | | |  |
| Abstract | 2 | See the PRISMA 2020 for Abstracts checklist. | Page 1-2; Line 1-34 |
| **INTRODUCTION** | | |  |
| Rationale | 3 | Describe the rationale for the review in the context of existing knowledge. | Page 3; Line 35–72 |
| Objectives | 4 | Provide an explicit statement of the objective(s) or question(s) the review addresses. | Page 3; Line 73–82 |
| **METHODS** | | |  |
| Eligibility criteria | 5 | Specify the inclusion and exclusion criteria for the review and how studies were grouped for the syntheses. | Page 4; Line 83–103 |
| Information sources | 6 | Specify all databases, registers, websites, organisations, reference lists and other sources searched or consulted to identify studies. Specify the date when each source was last searched or consulted. | Page 4; Line 84–92 |
| Search strategy | 7 | Present the full search strategies for all databases, registers and websites, including any filters and limits used. | Supplementary Table 2 |
| Selection process | 8 | Specify the methods used to decide whether a study met the inclusion criteria of the review, including how many reviewers screened each record and each report retrieved, whether they worked independently, and if applicable, details of automation tools used in the process. | Page 4–5; Line 104–123 |
| Data collection process | 9 | Specify the methods used to collect data from reports, including how many reviewers collected data from each report, whether they worked independently, any processes for obtaining or confirming data from study investigators, and if applicable, details of automation tools used in the process. | Page 4–5; Line 104–123 |
| Data items | 10a | List and define all outcomes for which data were sought. Specify whether all results that were compatible with each outcome domain in each study were sought (e.g. for all measures, time points, analyses), and if not, the methods used to decide which results to collect. | Page 5; Line 118–126 |
|  | 10b | List and define all other variables for which data were sought (e.g. participant and intervention characteristics, funding sources). Describe any assumptions made about any missing or unclear information. | Page 5; Line 118–126 |
| Study risk of bias assessment | 11 | Specify the methods used to assess risk of bias in the included studies, including details of the tool(s) used, how many reviewers assessed each study and whether they worked independently, and if applicable, details of automation tools used in the process. | Page 5; Line 127–142 |
| Effect measures | 12 | Specify for each outcome the effect measure(s) (e.g. risk ratio, mean difference) used in the synthesis or presentation of results. | Page 5; Line 143–148 |
| Synthesis methods | 13a | Describe the processes used to decide which studies were eligible for each synthesis (e.g. tabulating the study intervention characteristics and comparing against the planned groups for each synthesis (item #5)). | No |
|  | 13b | Describe any methods required to prepare the data for presentation or synthesis, such as handling of missing summary statistics, or data conversions. | Page 5–6; Line 149–157 |
|  | 13c | Describe any methods used to tabulate or visually display results of individual studies and syntheses. | No |
|  | 13d | Describe any methods used to synthesize results and provide a rationale for the choice(s). If meta-analysis was performed, describe the model(s), method(s) to identify the presence and extent of statistical heterogeneity, and software package(s) used. | Page 6; Line 158–169 |
|  | 13e | Describe any methods used to explore possible causes of heterogeneity among study results (e.g. subgroup analysis, meta-regression). | Page 6; Line 170–175 |
|  | 13f | Describe any sensitivity analyses conducted to assess robustness of the synthesized results. | Page 6; Line 176–182 |
| Reporting bias assessment | 14 | Describe any methods used to assess risk of bias due to missing results in a synthesis (arising from reporting biases). | No |
| Certainty assessment | 15 | Describe any methods used to assess certainty (or confidence) in the body of evidence for an outcome. | Page 6; Line 183–190 |
| **RESULTS** | | |  |
| Study selection | 16a | Describe the results of the search and selection process, from the number of records identified in the search to the number of studies included in the review, ideally using a flow diagram. | Page 7; Line 191–206 |
|  | 16b | Cite studies that might appear to meet the inclusion criteria, but which were excluded, and explain why they were excluded. | Page 7; Line 203–206 |
| Study characteristics | 17 | Cite each included study and present its characteristics. | Page 7–8; Line 207–220 |
| Risk of bias in studies | 18 | Present assessments of risk of bias for each included study. | Page 8; Line 221–236 |
| Results of individual studies | 19 | For all outcomes, present, for each study: (a) summary statistics for each group (where appropriate) and (b) an effect estimate and its precision (e.g. confidence/credible interval), ideally using structured tables or plots. | No |
| Results of syntheses | 20a | For each synthesis, briefly summarise the characteristics and risk of bias among contributing studies. | No |
|  | 20b | Present results of all statistical syntheses conducted. If meta-analysis was done, present for each the summary estimate and its precision (e.g. confidence/credible interval) and measures of statistical heterogeneity. If comparing groups, describe the direction of the effect. | Page 8–9; Line 237–268 |
|  | 20c | Present results of all investigations of possible causes of heterogeneity among study results. | No |
|  | 20d | Present results of all sensitivity analyses conducted to assess the robustness of the synthesized results. | Page 9; Line 269–273 |
| Reporting biases | 21 | Present assessments of risk of bias due to missing results (arising from reporting biases) for each synthesis assessed. | No |
| Certainty of evidence | 22 | Present assessments of certainty (or confidence) in the body of evidence for each outcome assessed. | Page 9; Line 274–280 |
| **DISCUSSION** | | |  |
| Discussion | 23a | Provide a general interpretation of the results in the context of other evidence. | Page 10–11; Line 281–330 |
|  | 23b | Discuss any limitations of the evidence included in the review. | Page 11; Line 331–345 |
|  | 23c | Discuss any limitations of the review processes used. | Page 11; Line 331–339 |
|  | 23d | Discuss implications of the results for practice, policy, and future research. | Page 11–12; Line 346–365 |
| **OTHER INFORMATION** | | |  |
| Registration and protocol | 24a | Provide registration information for the review, including register name and registration number, or state that the review was not registered. | Page 4; Line 93–95 |
|  | 24b | Indicate where the review protocol can be accessed, or state that a protocol was not prepared. | No |
|  | 24c | Describe and explain any amendments to information provided at registration or in the protocol. | No |
| Support | 25 | Describe sources of financial or non-financial support for the review, and the role of the funders or sponsors in the review. | Page 13; Line 418–420 |
| Competing interests | 26 | Declare any competing interests of review authors. | Page 13; Line 414–416 |
| Availability of data, code and other materials | 27 | Report which of the following are publicly available and where they can be found: template data collection forms; data extracted from included studies; data used for all analyses; analytic code; any other materials used in the review. | Page 13; Line 412–414 |

**Supplementary Table 2**: Search strategy.

| **Database** | **Keyword** | **Filter** | **Results** |
| --- | --- | --- | --- |
| PubMed | ("atrial fibrillation"[MeSH Terms] OR ("atrial"[All Fields] AND "fibrillation"[All Fields]) OR "atrial fibrillation"[All Fields] OR "a-fib"[All Fields] OR "af"[All Fields]) AND (("p"[All Fields] AND "wave"[All Fields] AND ("duration"[All Fields] OR "durations"[All Fields])) OR ("p-wave"[All Fields] AND ("duration"[All Fields] OR "durations"[All Fields])) OR "pwd"[All Fields] OR ("p"[All Fields] AND "wave"[All Fields] AND ("dispersability"[All Fields] OR "dispersable"[All Fields] OR "dispersal"[All Fields] OR "dispersals"[All Fields] OR "dispersant"[All Fields] OR "dispersants"[All Fields] OR "disperse"[All Fields] OR "dispersed"[All Fields] OR "disperser"[All Fields] OR "dispersers"[All Fields] OR "disperses"[All Fields] OR "dispersibilities"[All Fields] OR "dispersibility"[All Fields] OR "dispersible"[All Fields] OR "dispersing"[All Fields] OR "dispersion"[All Fields] OR "dispersions"[All Fields] OR "dispersities"[All Fields] OR "dispersity"[All Fields] OR "dispersive"[All Fields] OR "dispersively"[All Fields] OR "dispersivities"[All Fields] OR "dispersivity"[All Fields])) OR ("p-wave"[All Fields] AND ("dispersability"[All Fields] OR "dispersable"[All Fields] OR "dispersal"[All Fields] OR "dispersals"[All Fields] OR "dispersant"[All Fields] OR "dispersants"[All Fields] OR "disperse"[All Fields] OR "dispersed"[All Fields] OR "disperser"[All Fields] OR "dispersers"[All Fields] OR "disperses"[All Fields] OR "dispersibilities"[All Fields] OR "dispersibility"[All Fields] OR "dispersible"[All Fields] OR "dispersing"[All Fields] OR "dispersion"[All Fields] OR "dispersions"[All Fields] OR "dispersities"[All Fields] OR "dispersity"[All Fields] OR "dispersive"[All Fields] OR "dispersively"[All Fields] OR "dispersivities"[All Fields] OR "dispersivity"[All Fields])) OR "pwdis"[All Fields] OR ("p"[All Fields] AND "wave"[All Fields] AND ("parameter"[All Fields] OR "parameter s"[All Fields] OR "parameters"[All Fields])) OR ("p-wave"[All Fields] AND ("parameter"[All Fields] OR "parameter s"[All Fields] OR "parameters"[All Fields])) OR ("p"[All Fields] AND "wave"[All Fields] AND ("maximum"[All Fields] OR "maximums"[All Fields])) OR ("p-wave"[All Fields] AND ("maximum"[All Fields] OR "maximums"[All Fields])) OR ("p"[All Fields] AND "wave"[All Fields] AND ("minimum"[All Fields] OR "minimums"[All Fields])) OR ("p-wave"[All Fields] AND ("minimum"[All Fields] OR "minimums"[All Fields]))) AND ("stroke"[MeSH Terms] OR "stroke"[All Fields] OR "strokes"[All Fields] OR "stroke s"[All Fields]) | - | 265 |
| ProQuest | ("atrial fibrillation" OR "a-fib" OR af) in ABSTRACT AND  ("p wave dispersion" OR "p-wave dispersion" OR pdis OR pwdis OR "p wave duration" OR "p-wave duration" OR pwd OR ("p wave" NEAR/2 parameter*) OR ("p-wave" NEAR/2 parameter*) OR "p wave maximum" OR "p-wave maximum" OR "p wave minimum" OR "p-wave minimum") in ABSTRACT AND  (stroke OR strokes) in ANYWHERE | Peer reviewed | 265 |
| Wiley Online Library | "("atrial fibrillation" OR "a-fib" OR "AF")" anywhere and "("p wave dispersion" OR "p-wave dispersion" OR "pdis" OR "pwdis" OR "p wave duration" OR "p-wave duration" OR "pwd" OR "p wave maximum" OR "p-wave maximum" OR "p wave minimum" OR "p-wave minimum")" anywhere and "("stroke" OR "strokes")" anywhere | Journals | 117 |
| Google Scholar | allintitle: (“atrial fibrillation” OR “a-fib" OR “AF”) AND (“p wave dispersion” OR “p-wave dispersion” OR “pdis” OR “pwdis” OR “p wave duration” OR “p-wave duration” OR “pwd” OR “p wave maximum” OR “p-wave maximum” OR “p wave minimum” OR “p-wave minimum”) AND (“stroke”) | - | 176 |
| SAGE Journals | ("atrial fibrillation" OR "a-fib" OR "AF") AND ("p wave dispersion" OR "p-wave dispersion" OR "pdis" OR "pwdis" OR "p wave duration" OR "p-wave duration" OR "pwd" OR "p wave maximum" OR "p-wave maximum" OR "p wave minimum" OR "p-wave minimum") AND ("stroke") | - | 152 |
| EBSCOhost | AB ("atrial fibrillation" or "a-fib" or "af") AND AB ("p wave dispersion" OR "p-wave dispersion" OR "pdis" OR "pwdis" OR "p wave duration" OR "p-wave duration" OR "pwd" OR "p wave maximum" OR "p-wave maximum" OR "p wave minimum" OR "p-wave minimum") AND AB ("stroke") | Peer Reviewed | 95 |
| Europe PMC | (TITLE_ABS:("atrial fibrillation" OR "a-fib" OR "af")) AND (TITLE_ABS:("p wave dispersion" OR "p-wave dispersion" OR "pdis" OR "pwdis" OR "p wave duration" OR "p-wave duration" OR "pwd" OR "p wave maximum" OR "p-wave maximum" OR "p wave minimum" OR "p-wave minimum")) AND (TITLE_ABS:("stroke")) AND (SRC:MED OR SRC:PMC OR SRC:AGR OR SRC:CBA) NOT PUB_TYPE:"Review" |  | 198 |
| ScienceDirect | **TERMS:**  ("atrial fibrillation" OR "a-fib") AND ("p wave dispersion" OR "p wave duration" OR "p wave maximum" OR "p wave minimum" OR "p wave terminal force") AND ("stroke")  **TITLE, ABSTRACT, OR AUTHOR SPECIFIED KEYWORDS:**  ("p-wave dispersion" OR "p wave dispersion" OR "pwdis" OR "p wave duration" OR "p-wave duration" OR "p wave maximum" OR "p wave minimum") | Research article | 134 |
| Cochrane Library | #1 ("atrial fibrillation":ti,ab OR "a-fib":ti,ab OR AF:ti,ab)  #2 ("p wave dispersion":ti,ab OR "p-wave dispersion":ti,ab OR pdis:ti,ab OR pwdis:ti,ab OR "p wave duration":ti,ab OR "p-wave duration":ti,ab OR pwd:ti,ab OR ("p wave":ti,ab AND parameter*:ti,ab) OR ("p-wave":ti,ab AND parameter*:ti,ab) OR "p wave maximum":ti,ab OR "p-wave maximum":ti,ab OR "p wave minimum":ti,ab OR "p-wave minimum":ti,ab)  #3 (stroke:ti,ab)  #4 #1 AND #2 AND #3 | - | 14 |

**Supplementary Figure 1**: Sensitivity analysis using SMD in PWDur between post-CS NDAF and no AF groups.


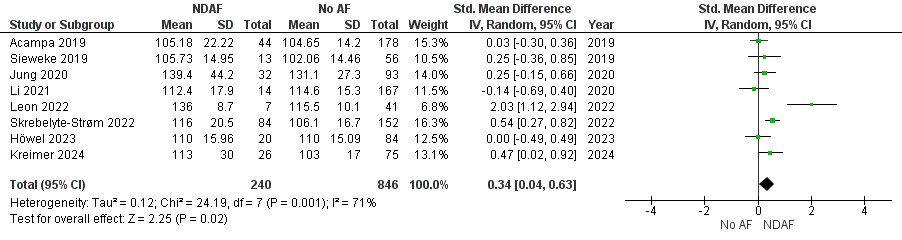


**Supplementary Figure 2**: Subgroup analysis of MD in PWDur based on age groups between post-CS NDAF and no AF groups.


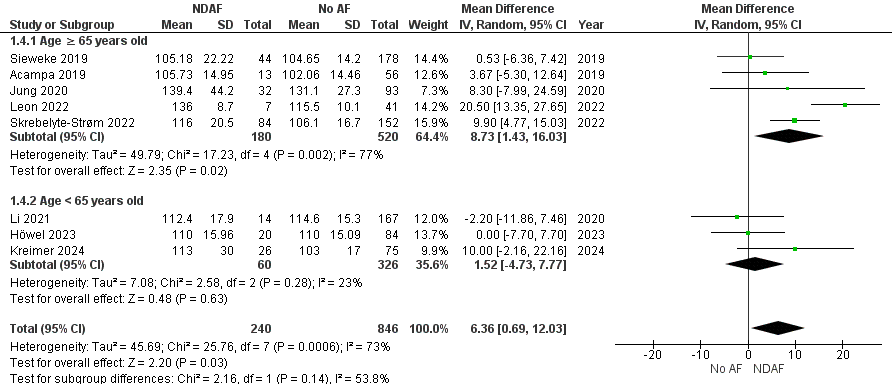


**Supplementary Figure 3**: Subgroup analysis of MD in PWDur based on male proportions between post-CS NDAF and no AF groups.


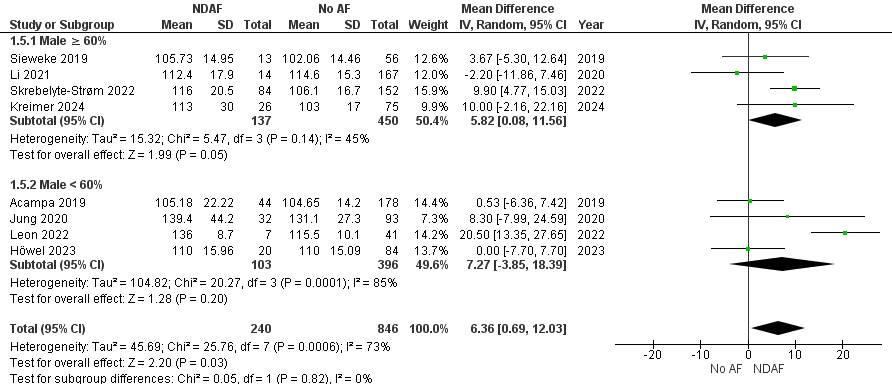


**Supplementary Figure 4**: Subgroup analysis of MD in PWDur based on follow-up duration between post-CS NDAF and no AF groups.


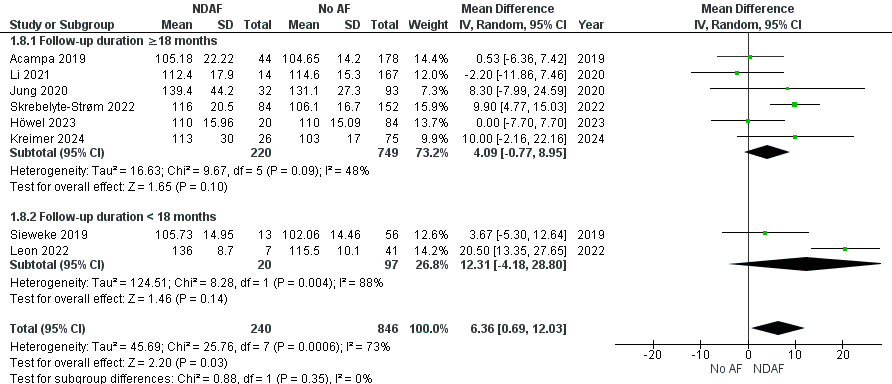


**Supplementary Figure 5**: Subgroup analysis of MD in PWDur based on demographic regions between post-CS NDAF and no AF groups.


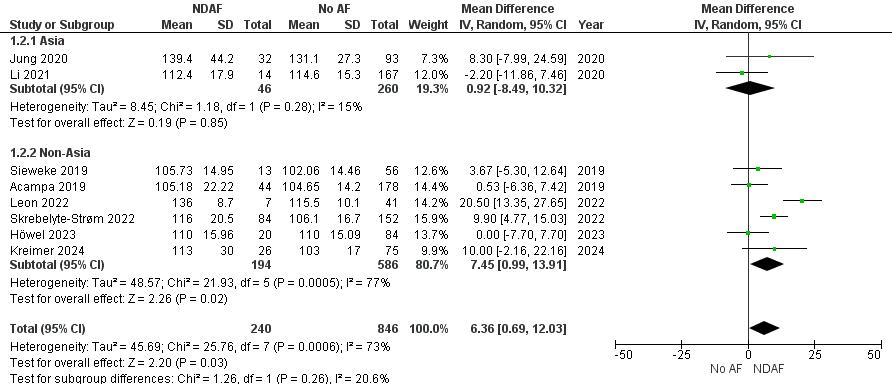


**Supplementary Figure 6**: Subgroup analysis of MD in PWDur based on HT percentages between post-CS NDAF and no AF groups.


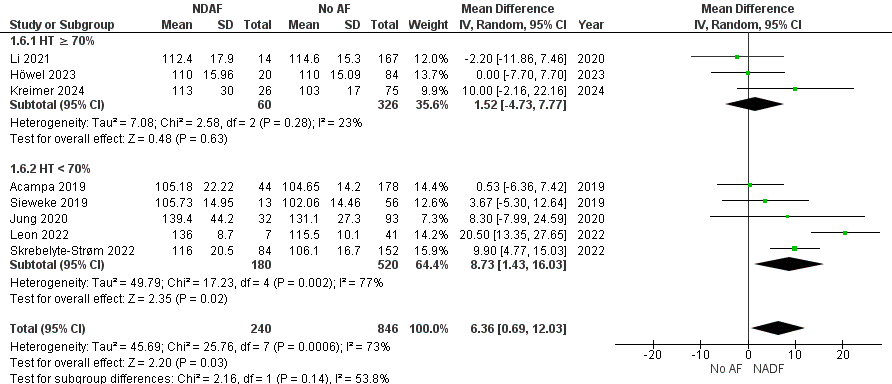


**Supplementary Figure 7**: Subgroup analysis of MD in PWDur based on DM percentages between post-CS NDAF and no AF groups.


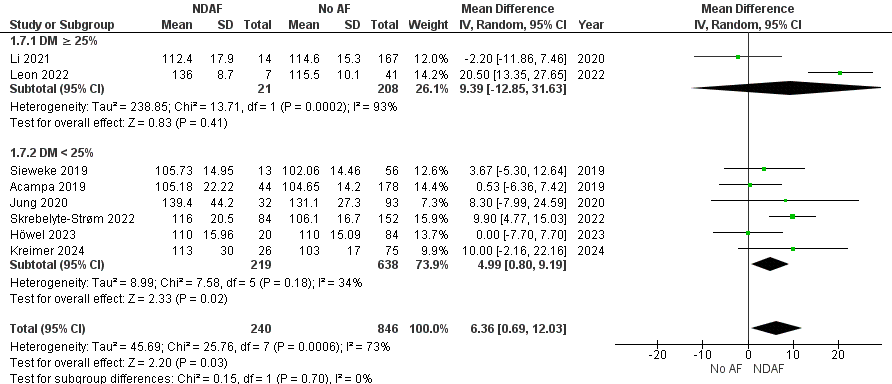


**Supplementary Figure 8**: Subgroup analysis of MD in PWDur based on AF definition between post-CS NDAF and no AF groups.

**
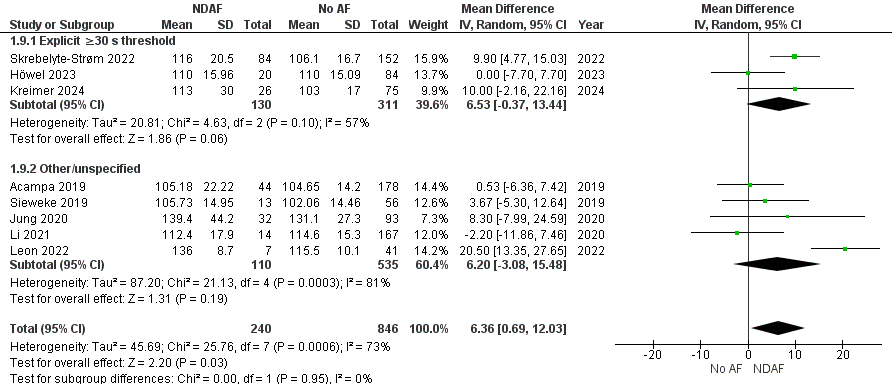
**

**Supplementary Figure 9**: Subgroup analysis of MD in PWDur based on AF detecion methods between post-CS NDAF and no AF groups.


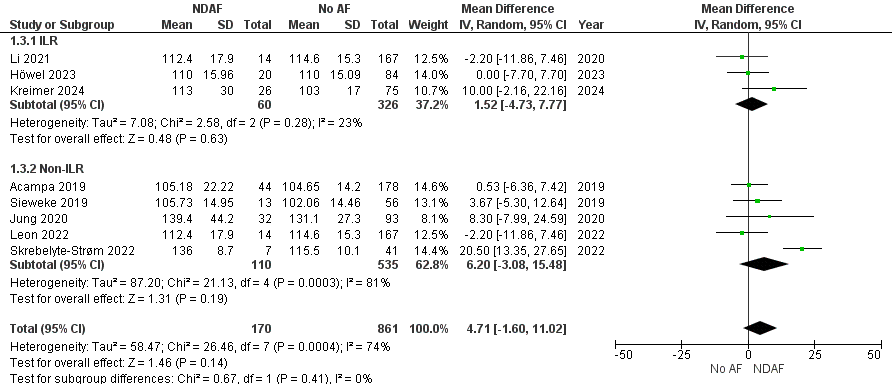


**Supplementary Figure 10**: Subgroup analysis of MD in PWDur based on ECG paper speed methods between post-CS NDAF and no AF groups.


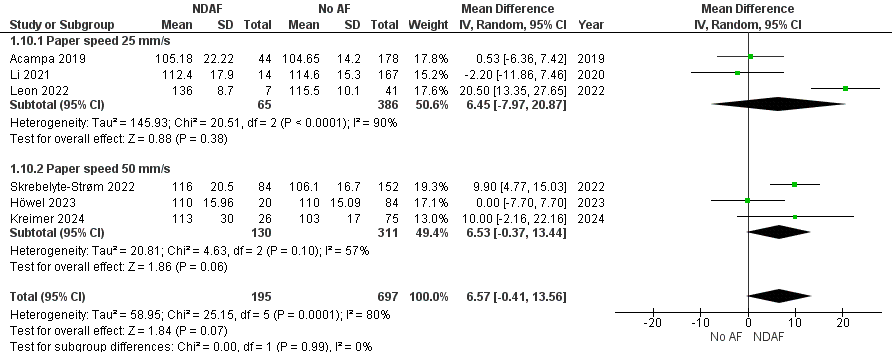


**Supplementary Figure 11**: Sensitivity analysis using SMD in PWDis between post-CS NDAF and no AF groups.


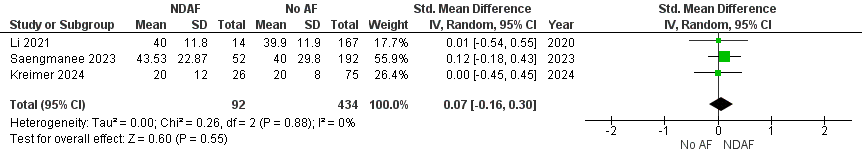

Supplement: Supplementary file 1 — Data S1: Supporting Information. [file JOA3-41-e70209-s001.docx]
